# Proceedings from the VIII International Symposium on Orofacial Clefts and Related Anomalies – Bauru Medical School, University of São Paulo (FMBRU-USP)

**DOI:** 10.1186/s12919-026-00401-4

**Published:** 2026-10-06

**Authors:** Bruno Mariano Ribeiro Braga, Vinicius Contrucci Dantas Segarra, Adélia Ferraz Daher Miranda, Ana Carla Pasquini Abu-Yaghi Nogueira, Ana Carolina Canuto Minozzi, Ana Laura Herrera Farha, Cristiane Sabino Vianna de Oliveira Domingues, Denicia Stefane Rodrigues Queiroz, Franciane da Silva Nascimento, Gabriel Bispo Botari, Gleisieli Baessa-Cardoso, Henrique Regonaschi Serigatto, Maria Carolina Neves, Nayara Tomazi Batista, Rosemery Aparecida Ferreira, Tatiane Aparecida da Silva, Verônica de Abreu Francisco, Marielena Layuno Matos, Christian Friederich Poets, Adriano Yacubian Fernandes, Cristiano Tonello, Ivy Kiemle Trindade-Suedam, Sergio Henrique Kiemle Trindade

**Affiliations:** 1 Bauru Medical School, University of São Paulo, Rua Silvio Marchione, 3-20 - Vila Nova Cidade Universitária, Bauru, São Paulo, 17012-900 Brazil; 2 Hospital for Rehabilitation of Craniofacial Anomalies, University of São Paulo, Bauru , São Paulo, Brazil; 3 Craniofacial Orthodontics, Seattle Children’s Hospital, Seattle, WA USA; 4 Craniofacial Center, Seattle Children’s Hospital, Seattle, WA USA; 5 Department of Orthodontics, School of Dental Medicine of the University of Puerto Rico Medical Science Campus, San Juan, Puerto Rico; 6 School of Dental Medicine, Ponce Health Sciences University, Ponce, Puerto Rico; 7 University of Tübingen, Tübingen, Germany; 8 Bauru School of Dentistry, University of São Paulo, Bauru, São Paulo, Brazil

**Keywords:** Cleft lip, Cleft palate, Craniofacial abnormalities, Pierre Robin sequence, Patience Care Team

## Abstract

The VIII International Symposium on Orofacial Clefts and Related Anomalies, organized by the Graduate Program in Rehabilitation Sciences at the Bauru Medical School, University of São Paulo (FMBRU-USP), brought together clinicians, researchers, students, and healthcare professionals to discuss recent advances in craniofacial rehabilitation. Held with the I FMBRU-USP Graduate Program Pre-Symposium, the scientific program comprised a keynote lectures on Robin Sequence management protocols, six thematic panels, and a poster session. The panels explored topics on Robin Sequence, digital technologies and artificial intelligence, velopharyngeal insufficiency, rehabilitation outcomes, translational research, and professional training. Across these themes, the symposium reinforced the value of multidisciplinary collaboration, evidence-based practice, emerging technologies, and international partnerships in advancing patient care and scientific innovation. This proceedings paper summarizes the principal discussions and key messages presented during the meeting, providing an overview of current advances, persistent challenges, and future directions in research, education, and the multidisciplinary management of individuals with orofacial clefts and related craniofacial anomalies.

## Background: the VIII International Symposium on Orofacial Clefts and Related Anomalies

Since its first edition in 2009, the Symposium has been organized by the Graduate Program in Rehabilitation Sciences at the Hospital for Rehabilitation of Craniofacial Anomalies, University of São Paulo (HRAC-USP), and has served as an important academic forum, bringing together clinicians, researchers, students, and healthcare professionals from Brazil and abroad. The International Symposium on Orofacial Clefts and Related Anomalies is a biennial scientific meeting dedicated to promoting knowledge exchange, interdisciplinary collaboration, and the dissemination of advances in the diagnosis, treatment, and rehabilitation of individuals with craniofacial anomalies. Over successive editions, the event has strengthened collaborations among institutions while fostering scientific discussion across multiple disciplines involved in craniofacial care.

The eighth edition marked an important milestone in the Symposium's history, taking place during a period of institutional transformation. Following the incorporation of HRAC-USP into Bauru Clinical Hospital (HC Bauru), the Graduate Program in Rehabilitation Sciences was transferred in 2025 to the Bauru Medical School, University of São Paulo (FMBRU-USP). In the same year, the Program achieved a CAPES (Coordenação de Aperfeiçoamento de Pessoal de Nível Superior) grade 6 out of 7, the Brazilian federal agency responsible for regulating and funding graduate education, recognizing its excellence, scientific productivity, and internationalization. To mark this new phase, the organizing committee introduced the First Pre-Symposium of the FMBRU-USP Graduate Program. The initiative showcased the newly established research area, Interdisciplinary Care Across the Life Cycles, allowing faculty advisors to present their research lines while fostering the integration of newly admitted master's and PhD students into the Program before the main scientific meeting.

While maintaining its traditional emphasis on orofacial clefts and related craniofacial anomalies, the scientific program expanded to encompass related fields, including craniofacial growth and development, digital technologies, artificial intelligence (AI), regenerative medicine, surgical innovation, imaging, dentistry, speech-language pathology, genetics, and multidisciplinary patient-centered care. This broader scope reinforced the Symposium's role as a platform for integrating contemporary research and clinical practice across the full spectrum of craniofacial health.

## Symposium overview

**Fig. 1:**
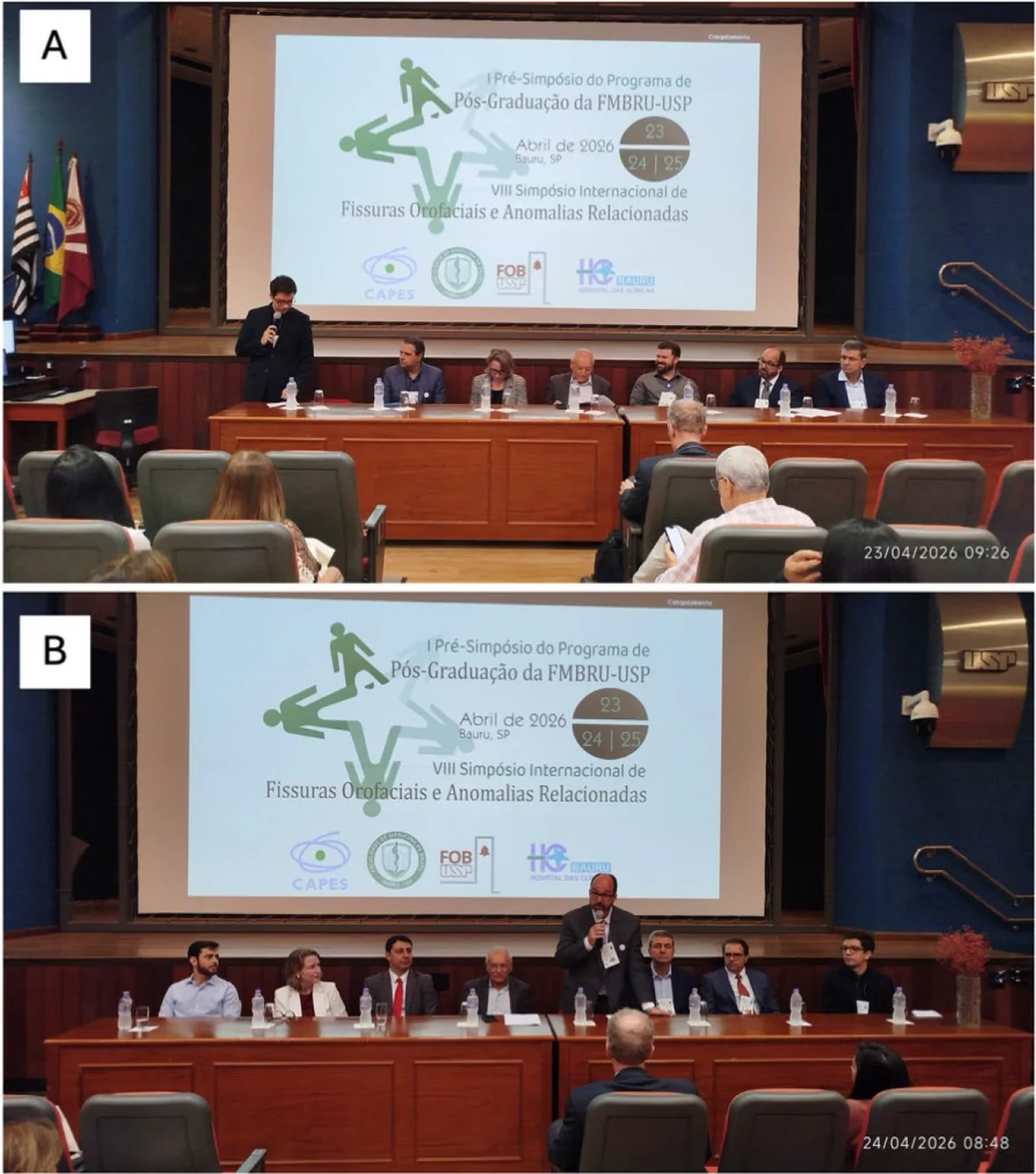
Opening ceremony of the VIII International Symposium on Orofacial Clefts and Related Anomalies. **A** PhD student and student representative, Vinicius Contrucci Dantas Segarra, speaking during the opening ceremony. **B** Professor Sergio Henrique Kiemle Trindade, faculty member and Coordinator of the Graduate Program in Rehabilitation Sciences (FMBRU-USP), speaking during the opening ceremony

The symposium was structured across three days, combining the I Pre-Symposium of the FMBRU-USP Graduate Program (April 23) with the VIII International Symposium on Orofacial Clefts and Related Anomalies (April 24–25) (Table 1) (Fig. 1). Both events were planned to highlight the relevance of the different fields involved in the treatment of craniofacial anomalies and their interrelationships. All FMBRU-USP graduate program advisors, along with international researchers, presented their research lines and associated clinical expertise related to their research areas. The agenda included keynote lectures, thematic panel discussions, round tables, and a poster session, making clear the breadth and interdisciplinary nature of the field.

**Table 1:** Symposium scientific program

	**Activity**
**Day 1 (Thursday)**	Opening remarks
	I Pre-Symposium of the FMBRU-USP Graduate Program
**Day 2 (Friday)**	Main Event: VIII International Symposium on Orofacial Clefts and Related Anomalies: Keynote Lectures: 1) The University Of Tübingen Protocol For Robin Sequence—Tübingen Palatal Plate (TPP) (Speaker: Prof. Dr. Christian Poets) 2) Seattle Children’s Hospital Treatment Protocol For Patients With Robin Sequence (Speaker: Prof. Dr. Marielena Layuno Matos)
	Panel 1/Roundtable: Treatment of Robin Sequence
	Panel 2: Dentistry and New Technologies in Craniofacial Anomalies
	Panel 3: Treatment of Velopharyngeal Insufficiency
	Panel 4: Evaluation of Rehabilitation Outcomes
**Day 3 (Saturday)**	Panel 5: Interdisciplinary and Translational Research in Craniofacial Anomalies
	Panel 6: Internationalization as a Driver of Scientific Excellence in Craniofacial Rehabilitation
	Poster Session Presentation
	Closing remarks

## Opening remarks

The symposium opened with an institutional ceremony attended by Prof. Ivy Kiemle Trindade-Suedam on behalf of USP Office of Graduate Studies, (PRPG-USP), Prof. José Sebastião dos Santos (Dean of FMBRU-USP), Prof. Rodrigo Cardoso de Oliveira (Dean of FOB-USP), Prof. Júlio César Garcia de Alencar (Director of HC Bauru), Prof. Sergio Henrique Kiemle Trindade (Coordinator of the Graduate Program in Rehabilitation Sciences), Prof. Adriano Yacubian Fernandes (Vice-Coordinator of the Graduate Program in Rehabilitation Sciences), and Vinicius Contrucci Dantas Segarra (student representative). In his opening remarks, Prof. dos Santos emphasized that the Graduate Program in Rehabilitation Sciences has been essential in strengthening FMBRU-USP by increasing scientific production, expanding national and international collaborations, and enhancing the institution's visibility in both clinical care and research.

Following the opening ceremony, Prof. Ivy Kiemle Trindade-Suedam opened the scientific program with the presentation *“HRAC-USP Graduate Program: Where We Came from and Where We Are Going? How to Consolidate a Research Line,”* marking the 25-year trajectory of the Graduate Program in Rehabilitation Sciences. The presentation addressed the program’s evolution, scientific achievements, and the consolidation of research lines in craniofacial rehabilitation, highlighting the importance of continuity, collaboration, innovation, and long-term planning. The recent CAPES grade of 6 out of 7 was highlighted reflecting the program’s scientific productivity, internationalization, and contribution to graduate education.

## Keynote lectures

### 1) The University of Tübingen protocol for Robin Sequence—Tübingen Palatal Plate (TPP)

Speaker: Prof. Dr. Chistian Poets, MD (University of Tübingen, Germany).

**Fig. 2:**
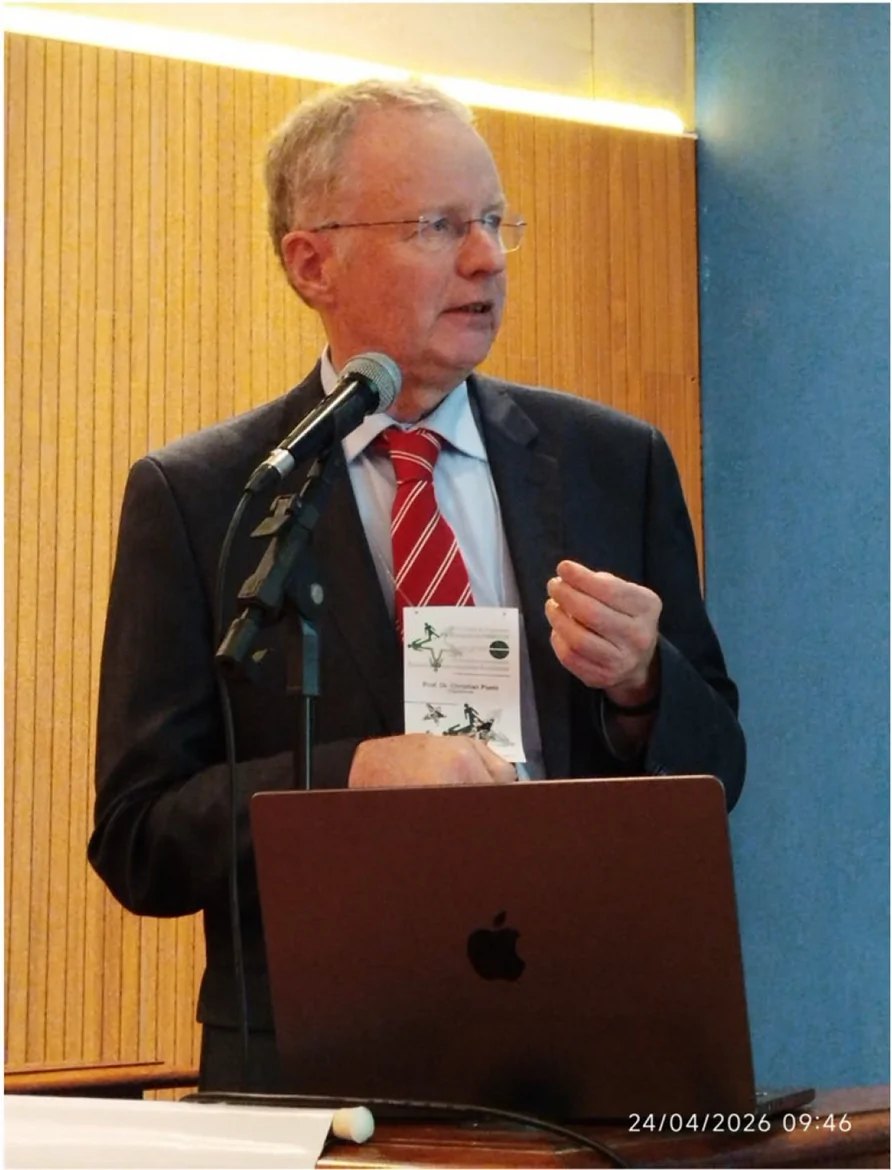
Prof. Poets presenting the Tübingen Palatal Plate experience at Tübingen University Hospital during the VIII International Symposium on Orofacial Clefts and Related Anomalies, FMBRU-USP

Dr. Christian Friedrich Poets is a distinguished Professor in the field of neonatology and sleep medicine and Head of the Department of Neonatology at the University Hospital Tübingen in Germany. He presented the Tübingen Palatal Plate (TPP), developed by his team, as a treatment for upper airway obstruction in infants with Robin Sequence. The device consists of a palatal plate with an individualized velar extension to reposition the tongue base, thereby relieving airway obstruction while avoiding more invasive interventions such as tracheostomy or mandibular distraction osteogenesis. Prof. Poets presented highly favorable long-term outcomes from the Tübingen experience, and the protocol has since been adopted by several centers worldwide. The device requires careful customization, specialized follow-up adjustments, because of the complexity of upper airway anatomy, particularly in infants with syndromic Robin Sequence [1, 2] (Fig. 2).

### 2) Seattle Children’s Hospital treatment protocol for patients with Robin Sequence

Speaker: Prof. Dr. Marielena Layuno-Matos, DMD (Craniofacial Orthodontist—University of Porto Rico).

**Fig. 3:**
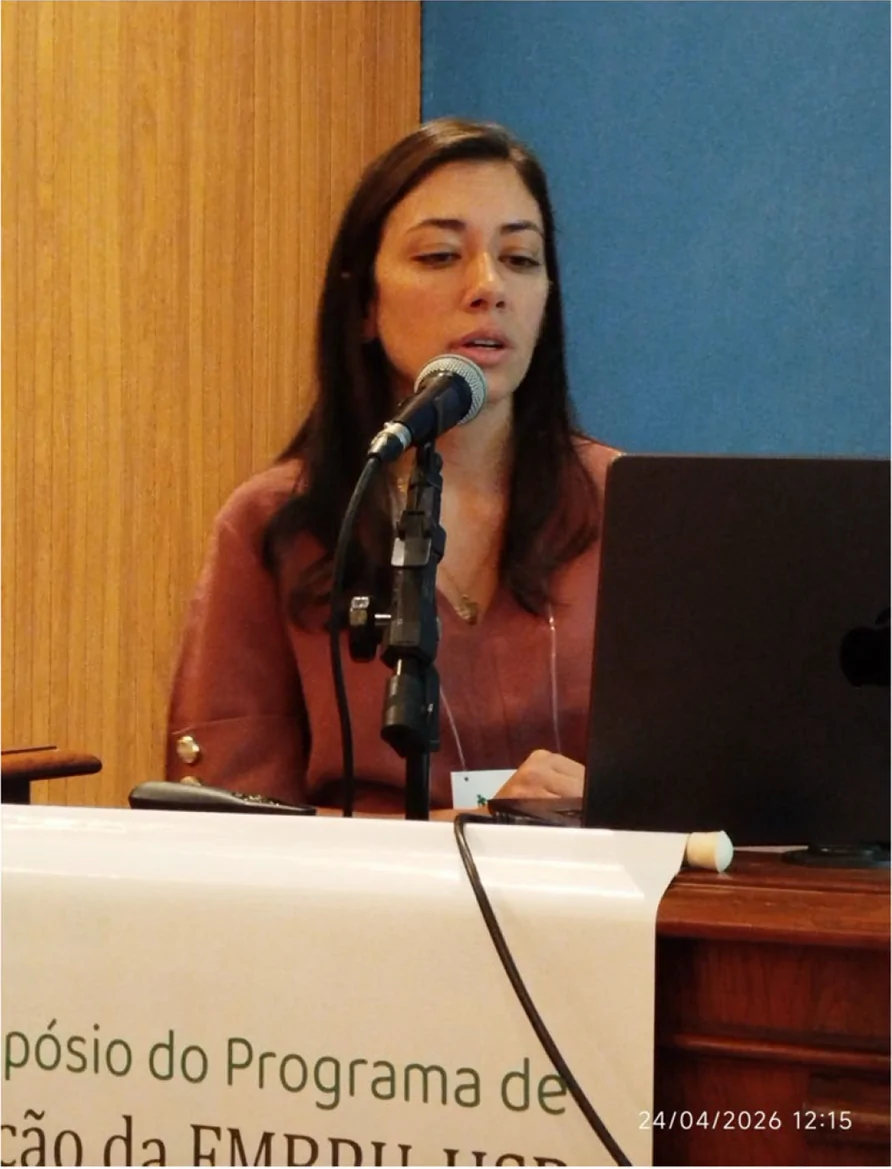
Dr. Layuno-Matos presenting the Seattle Children's Hospital Treatment Protocol for Robin Sequence during the VIII International Symposium on Orofacial Clefts and Related Anomalies

Professor Dr. Layuno-Matos is a board-certified orthodontist who completed specialized training in craniofacial orthodontics at Seattle Children's Hospital (SCH), Washington, United States of America. She presented on the management protocols used in SCH for patients with Robin Sequence, highlighting the use of the Orthodontic Airway Plate (OAP) as a nonsurgical treatment to relieve tongue-based upper airway obstruction. Initial management consists of conservative measures, including side- and prone-positioning, nasogastric feeding, and respiratory support. Historically, for patients who required escalation in care, surgical intervention, such as mandibular distraction osteogenesis or tracheostomy, was considered. In August 2023, OAP therapy was introduced at SCH; inspired by the Tübingen Palatal Plate, this appliance displaces the base of the tongue forward and maintains upper airway patency. The institution's initial clinical experience with the OAP was shown through case examples, emphasizing on protocols and workflows involving interdisciplinary collaboration with otolaryngology, craniofacial orthodontics, feeding specialists, bioengineering, developmental therapists, and nursing [3, 4] (Fig. 3).

## Panel 1: treatment of Robin Sequence

The first panel focused on current treatment strategies and innovations for Robin Sequence (RS), highlighting multidisciplinary protocols adopted by rehabilitation centers in Tübingen (Germany), Seattle (USA), and Bauru (Brazil). In this panel, it was discussed the Tübingen palatal plate (TPP) and Orthodontic Airway Plate (OAP), an intraoral device designed to relieve upper airway obstruction by repositioning the tongue base [1, 2]. Although its effectiveness has led to increasing adoption in specialized centers, its limited availability means that alternative conservative approaches remain essential. To address the respiratory distress, non-invasive techniques such as the prone position are used in some cases, providing immediate, although not long-lasting effects. Other methods include the nasopharyngeal cannula, which is more effective than the prone position. [5], with treatment guided by objective assessment through polysomnography, which was presented by Prof. Dr. Sergio H. K. Trindade, to quantify airway obstruction and monitor outcomes. When conservative approaches fail or severe airway obstruction is present, surgical management becomes necessary. Mandibular distraction osteogenesis was highlighted by Prof. Dr. Cristiano Tonello, as the preferred surgical option, because it provides rapid improvement in both airway patency and feeding [6]. In contrast, glossopexy is currently less favored and should only be considered after careful multidisciplinary discussion. Tracheostomy remains an option for the most severe cases when other interventions are unsuccessful. The panel also emphasized the importance of long-term follow-up after surgical interventions and the routine genetic evaluation, because approximately 50% of the cases are syndromic, i.e., associated with other anomalies. The distinction between isolated and syndromic RS is crucial to determining the steps of the rehabilitation process, since the interventions may differ, as well as providing accurate genetic counseling [6, 7].

### Panel highlights

This panel was presented by Prof. Dr. Christian Poets, Prof. Dr. Cristiano Tonello, Prof. Dr. Isabel Cristina Drago Marquezini Salmen, Prof. Dr. Marielena Layuno Matos, Prof. Dr. Sergio Henrique Kiemle Trindade, and Prof. Dr. Roseli Maria Zechi-Ceide. Robin Sequence requires individualized, multidisciplinary management based on the severity of upper airway obstruction. Treatment ranges from conservative approaches to surgical interventions, with the TPP and OAP emerging as an innovative, minimally invasive option for selected patients. Objective airway assessment and long-term follow-up are essential to optimize clinical outcomes.

## Panel 2: dentistry and new technologies in craniofacial anomalies

This panel explored how digital technologies have enhanced pediatric dental care for patients with cleft lip and palate by supporting clinical documentation, objective assessment, and interdisciplinary treatment planning. Recent advances include digital intraoral scanning, three-dimensional (3D) assessment of dental arches and craniofacial growth, and stereophotogrammetry for longitudinal facial analysis [8, 9]. As craniofacial growth approaches completion, orthognathic surgery becomes an essential component of comprehensive rehabilitation for patients with cleft lip and palate presenting residual skeletal discrepancies, requiring individualized interdisciplinary assessment. The panel also discussed early orthognathic intervention in selected adolescents, balancing potential functional, esthetic, and psychosocial benefits against the risks associated with residual craniofacial growth, relapse, and the potential need for retreatment [10, 11]. Complementing these advances, AI is increasingly supporting diagnosis, treatment planning, and outcome assessment in cleft care. AI-based approaches have been developed for automated cleft morphology [12] and occlusion classification [13], as well as 3D image segmentation and reconstruction and objective evaluation of alveolar bone graft outcomes, with the potential to improve diagnostic accuracy, prediction of treatment outcomes, and the reproducibility of quantitative analyses [14].

### Panel highlights

This panel was presented by Prof. Dr. Daniela Garib, Prof. Dr. Felicia Miranda, Prof. Dr. Maria Aparecida de Andrade Moreira Machado, Prof. Dr. Renato Yassutaka Faria Yaedú, Prof. Dr. Terumi Okada Ozawa, and Prof. Dr. Thais Marchini de Oliveira Valarelli, who highlighted the integration of digital technologies and AI into craniofacial rehabilitation. Discussions emphasized digital workflows for longitudinal follow-up, interdisciplinary treatment planning, and objective outcome assessment. Advances in AI were presented as tools to improve diagnosis, image analysis, surgical planning, and treatment outcome evaluation, supporting more precise and personalized patient care.

## Panel 3: treatment of velopharyngeal insufficiency

This panel explored complementary approaches to the assessment and rehabilitation of velopharyngeal insufficiency (VPI), highlighting the integration of instrumental evaluation, surgical management, and speech-language therapy. The pressure-flow technique was presented as an objective method for assessing velopharyngeal function by indirectly estimating the velopharyngeal orifice area during speech production. The technique provides quantitative measurements that support the interpretation of velopharyngeal function and contribute to clinical decision-making [15]. Surgical management at HRAC-USP is guided by perceptual-auditory and instrumental assessments, together with the size of the velopharyngeal gap, closure pattern, and the mobility of the soft palate and pharyngeal walls [16, 17]. Treatment of VPI is individualized according to velar anatomy and function, gap size, and lateral pharyngeal wall movement, assessed mainly by nasopharyngoscopy and videofluoroscopy. Palate re-repair with radical intravelar veloplasty is preferred when levator abnormalities or velar asymmetry are present. For persistent VPI with adequate palatal function, posterior pharyngeal wall augmentation may be used for small gaps, while a superiorly based pharyngeal flap is indicated for larger gaps with adequate lateral wall movement. At HRAC-USP, flap design is tailored to individual anatomy and function to optimize closure while minimizing respiratory complications [16, 18]. For patients who are not candidates for surgery, rehabilitation may be achieved with a palatal prosthesis incorporating a pharyngeal bulb, developed through collaboration between speech-language pathologists and dental professionals to reduce the velopharyngeal gap. Successful rehabilitation also depends on targeted speech therapy to optimize velopharyngeal function and speech outcomes [19, 20]. Overall, the panel emphasized that effective management of velopharyngeal insufficiency requires individualized treatment planning based on comprehensive assessment and close collaboration among different specialties to achieve optimal functional outcomes.

### Panel highlights

This panel was presented by Prof. Dr. Ana Paula Fukushiro, Prof. Dr. Maria Inês Pegoraro Krook and Prof. Dr. Nivaldo Alonso. Individualized management of velopharyngeal insufficiency relies on objective assessment, multidisciplinary decision-making, and tailored rehabilitation strategies. Instrumental evaluation, appropriate surgical selection, and prosthetic rehabilitation combined with speech therapy are essential to optimize velopharyngeal function and speech outcomes.

## Panel 4: evaluation of rehabilitation outcomes

This panel emphasized the importance of clinical and translational research in evaluating rehabilitation outcomes and continuously improving the management of individuals with orofacial clefts. At HRAC-USP long-term institutional research and participation in international multicenter collaborations have generated robust evidence to refine treatment protocols and optimize patient care. The discussion highlighted the contribution of the Timing of Primary Surgery for Cleft Palate (TOPS) trial, which demonstrated that children who underwent palatoplasty at 6 months of age exhibited a higher frequency of canonical babbling at 12 months and a lower prevalence of velopharyngeal insufficiency at 5 years in the primary analysis. Regarding dentofacial outcomes, no clinically meaningful differences were observed between the groups [21]. The Florida Project, developed at HRAC-USP, evaluated institutional outcomes while incorporating training in international surgical techniques. Its findings showed that techniques promoting more effective muscle reconstruction achieve excellent speech and velopharyngeal function, although potential effects on facial growth must also be considered. As these cohorts have now reached adulthood, long-term functional, structural, and aesthetic outcomes can be comprehensively assessed [22–24]. The panel also highlighted the increasing role of stereophotogrammetry as a non-invasive, 3D technology for objectively evaluating facial morphology, comparing surgical protocols, and monitoring craniofacial growth over time. By providing accurate quantitative measurements, this technology supports evidence-based assessment of rehabilitation outcomes and reduces the subjectivity of conventional facial analysis. The integration of long-term clinical research with emerging imaging technologies is transforming the evaluation of rehabilitation outcomes, enabling more precise and evidence-based approaches to patient care.

### Panel highlights

This panel was presented by Prof. Dr. Renata Paciello Yamashita, Prof. Dr. Simone Soares and Prof. Dr. Jennifer de Cássia Rillo Dutka. Longitudinal clinical research and international collaborations provide the evidence needed to refine cleft rehabilitation protocols. Advanced technologies such as stereophotogrammetry enable objective outcome assessment, supporting individualized, evidence-based decisions that balance function, facial growth, and aesthetics.

## Panel 5: interdisciplinary and translational research in craniofacial anomalies

This panel explored interdisciplinary and translational research approaches that are expanding the understanding of craniofacial anomalies through the integration of neurophysiology, biochemistry, molecular biology, genetics, and neuroimaging. Advances in the neurophysiology of hearing highlighted the importance of functional assessment for understanding auditory outcomes in affected individuals [25]. The application of proteomics was presented as an emerging strategy for investigating craniofacial anomalies, demonstrating how protein profiling captures the combined effects of genetic and environmental factors [26]. Recent developments in molecular genetics have also transformed the investigation of craniofacial anomalies, with the next-generation sequencing (NGS) methodologies, enabling a more comprehensive characterization of the genetic basis of these conditions [27]. The integration of multi-omics data with detailed phenotypic characterization was emphasized as essential for improving the understanding of genotype–phenotype relationships. The complexity of craniofacial disorders was further illustrated by conditions such as craniofacial microsomia, which presents heterogeneous clinical manifestations and genetic variants [28]. The complementary role of computed tomography with 3D reconstruction and magnetic resonance imaging was also highlighted, particularly in the evaluation of syndromic craniosynostoses, where comprehensive characterization of craniofacial and intracranial abnormalities supports individualized treatment planning [29].

### Panel highlights

This panel was presented by Prof. Dr. Adriano Yacubian Fernandes, Prof. Dr. Kátia de Freitas Alvarenga, Prof. Dr. Lucimara Teixeira das Neves, Prof. Dr. Marília Afonso Rabelo Buzalaf, and Prof. Dr. Siulan Vendramini Paulovich-Pittoli. Interdisciplinary and translational research is advancing the understanding of craniofacial anomalies through the integration of functional assessment, multi-omics technologies, genetics, and advanced imaging. Combining these complementary approaches supports more accurate diagnosis, genotype–phenotype characterization, precision medicine, and individualized patient care.

## Panel 6: internationalization as a driver of scientific excellence in craniofacial rehabilitation

The panel addressed the growing importance of graduate education and internationalization in fostering scientific excellence. Academic mobility, institutional partnerships, researcher exchange, and multicenter collaborations were presented as opportunities to strengthen research capacity, standardized clinical protocols, and accelerate the translation of scientific discoveries into clinical practice. The role of research funding was emphasized as essential for supporting scholarships, research infrastructure, innovation, and international collaboration, ensuring the continuity of high-quality teaching and scientific production. Together, these initiatives illustrate how investment in education, collaboration, and research creates a sustainable framework for developing future leaders while advancing the quality and global impact of craniofacial rehabilitation [30, 31].

### Panel highlights

This panel was presented by Prof. Dr. Carlos Ferreira dos Santos. Integrating interdisciplinary education, international partnerships, and research funding strengthens workforce development and accelerates innovation, improving both patient care and scientific advancement.

## Poster presentation session and awards

**Fig. 4:**
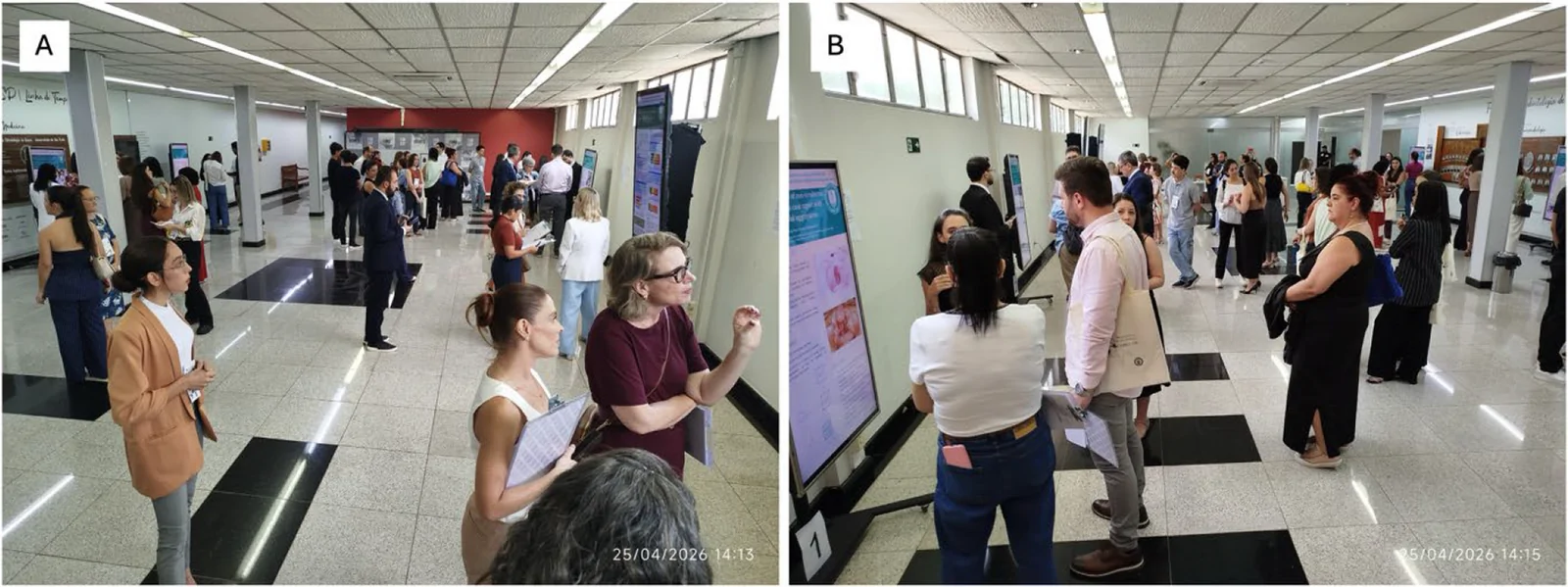
**A-B** Poster presentation session at the VIII International Symposium on Orofacial Clefts and Related Anomalies, FMBRU-USP

The VIII edition received 90 abstract submissions, of which 84 were accepted for poster presentation (Fig. 4) and distributed across three thematic categories: a) original research; b) case reports; and c) systematic reviews. Awards were granted to the highest-ranked presentations in each category.

### Original research best presentations

- A Longitudinal Assessment of Velopharyngeal Dysfunction in Patients with Unilateral Cleft Lip and Palate. *Authors: Amanda Herrera Farha, Jeniffer de Cássia Rillo Dutka, Olívia Mesquita Vieira de Souza. Mentor: Maria Inês Pegoraro-Krook.*
- AI-Powered 3D Workflow for Alveolar Bone Grafting and Mandibular Symphysis Donor Site Outcomes. *Authors: Bruno Mariano Ribeiro Braga, Claudia Resende Leal, Roberta Martinelli Carvalho, Marilia Yatabe, Cristiano Tonello, Rodrigo Teixeira and Lucia Cevidanes. Mentor: Terumi Okada Ozawa.*
- Identification of Speech Hypernasality by Lay Listeners and Perception of Prejudice. *Authors: Denicia Stefane Rodrigues Queiroz, Julia Bernasconi Braga, Amanda Herrera Farha, Olívia Mesquita Vieira de Souza, Jennifer de Cássia Rillo Dutka. Mentor: Maria Inês Pegoraro-Krook.*

#### Honorable mention

- Analysis of Masticatory Function and Its Relationship with the Nutritional Status of Individuals with Treacher Collins Syndrome. *Authors: Laís Hollara Medeiros, Leide Vilma Fidelis da Silva, Patricia Martins Bueno, Ana Paula Fukushiro. Mentor: Ivy Kiemle Trindade-Suedam.*

### Case report best presentations

- Sleep-Disordered Breathing Findings in an Adult with Repaired Cleft Lip and Palate and Pharyngeal Flap: A Case Report. *Authors: Beatriz Garrocini Torres, Bruna Mara Adorno Marmontel Araújo, Sergio Henrique Kiemle Trindade. Mentor: Ana Paula Fukushiro.*
- Velopharyngeal Function Changes After Maxillary Advancement Without Speech Complaints: A Case Report. *Authors: Franciane da Silva Nascimento, Barbara Souza dos Santos. Mentor: Renata Paciello Yamashita.*

#### Honorable mention

- Influence of Rapid Maxillary Expansion on the Respiratory Event Index: A Case Report. *Authors: Ana Caroline da Silva Pinto, João Gabriel Rando Poiani, Sergio Henrique Kiemle Trindade, Felicia Miranda, Camila Massaro, Daniela Garib. Mentor: Rodrigo Teixeira.*

### Systematic review best presentations

- Metacognition and Simulation for the Development of Clinical Reasoning and Training for the Care of Craniofacial Anomalies. *Authors: Felipe Oliveira Silva, Diego Andrés Díaz-Guio, Raphael Raniere de Oliveira Costa. Mentor: Alessandra Mazzo.*

#### Honorable mention

- Endocrine Disorders in Patients with Cleft Lip and Palate: A Systematic Review and Implications for Preoperative Evaluation. *Authors: Marina Oliveira Alves, Quezia Maria Sabino Andrade, Verônica Francisco. Mentor: Fernanda Leite.*

## Closing remarks

The Symposium’s final session was dedicated to honors and acknowledgments. Former coordinators of the Graduate Program in Rehabilitation Sciences, including Prof. Inge Elly Kiemle Trindade (2003–2011), Prof. Daniela Gamba Garib Carreira (2011–2017), Prof. Ana Paula Fukushiro (2017–2019), Prof. Jeniffer de Cassia Rillo Dutka (2021–2022) and Prof. Ivy Kiemle Trindade-Suedam (2019–2021; 2022–2025), were recognized for their contributions to the development and consolidation of the program. Special recognition was given to Prof. Inge Elly Kiemle Trindade, founder of the Graduate Program, for her pioneering role in establishing its academic foundation and contributing to its long-term trajectory. The ceremony also acknowledged the contributions of the Graduate Program administrative staff and technical-administrative employees for their support in maintaining academic activities. Reflecting these institutional developments, the Symposium was organized through a collaborative effort involving the University of São Paulo (USP), USP Office of Graduate Studies (PRPG-USP), CAPES, FMBRU-USP, FOB-USP, and HC Bauru.

## Impact and engagement

Over its successive editions, the event has accumulated more than 500 abstract submissions and drawn over 1,000 attendees in total. The most recent edition brought together 200 participants, including undergraduate and graduate students, as well as professionals in the field. The participation of international speakers further enhanced the Symposium by introducing innovative therapeutic and diagnostic approaches in craniofacial anomaly management to the local academic environment. Sponsoring companies took part directly in the event, presenting their products and facilities to attendees and fostering interaction between industry and the academic community.

As HRAC-USP, an institution with more than 50 years of history in multidisciplinary care and rehabilitation of craniofacial anomalies, becomes incorporated into the HC Bauru structure, maintaining its academic and clinical legacy remains a priority. Preserving its tradition in research, teaching, and multidisciplinary craniofacial care will be essential to ensure that this expertise continues to contribute to future developments within the evolving institutional framework.

## Data Availability

The data that support the findings of this study are not deposited in any public repository. However, they may be made available from the corresponding author upon reasonable request and with appropriate institutional and ethical approvals. The abstracts presented at the symposium will be made available through the University of São Paulo institutional repository.

## Abbreviations

3D: Three-dimensional
AI: Artificial intelligence
CAPES: Coordenação de Aperfeiçoamento de Pessoal de Nível Superior
FMBRU-USP: Bauru Medical School, University of São Paulo
FOB-USP: Bauru School of Dentistry, University of São Paulo
HC Bauru: Bauru Clinical Hospital
HRAC-USP: Hospital for Rehabilitation of Craniofacial Anomalies, University of São Paulo
NGS: Next-generation sequencing
OAP: Orthodontic Airway Plate
PROAP: Programa de Apoio à Pós-Graduação (Graduate Program Support Program)
PRPG-USP: University of São Paulo Office of Graduate Studies
RS: Robin Sequence
SCH: Seattle Children's Hospital
TOPS: Timing of Primary Surgery for Cleft Palate (trial)
TPP: Tübingen Palatal Plate
USP: University of São Paulo
VPI: Velopharyngeal insufficiency
